# Validity and Wear Compliance of Wrist-Worn Consumer Activity Trackers Among Japanese School-Aged Children Under Free-Living Conditions

**DOI:** 10.3390/children13020184

**Published:** 2026-01-28

**Authors:** Mitsuya Yamakita, Daisuke Ando, Miri Sato, Yuka Akiyama, Kaori Yamaguchi, Zentaro Yamagata

**Affiliations:** 1 Faculty of Nursing, Yamanashi Prefectural University, 1-6-1 Ikeda, Kofu 400-0062, Japan; 2 Center for Birth Cohort Studies, Graduate Faculty of Interdisciplinary Research, University of Yamanashi, Kofu 400-0016, Japan; akiyama.yuka@c2c.ac.jp (Y.A.); yamagata-z@ncchd.go.jp (Z.Y.); 3 Division of Human Sciences, Faculty of Education, Graduate Faculty of Interdisciplinary Research, University of Yamanashi, Kofu 400-0016, Japan; dandoh@yamanashi.ac.jp; 4 Department of Health and Psychosocial Medicine, School of Medicine, Aichi Medical University, Nagakute 480-1195, Japan; mirimirimiri37@yahoo.co.jp; 5 Faculty of Health and Nutrition, Yamanashi Gakuin University, Kofu 400-8575, Japan; 6 Institute of Health and Sport Sciences, University of Tsukuba, Tsukuba 305-8574, Japan; yamaguchi.kaori.gf@u.tsukuba.ac.jp; 7 Think Tank for Children and Parents, National Center for Child Health and Development, Tokyo 157-8535, Japan

**Keywords:** wearable electronic devices, accelerometry, exercise, sedentary time, acceptability

## Abstract

**Highlights:**

**What are the main findings?**

• The wrist-worn consumer activity trackers showed strong correlations with a waist-worn accelerometer for step count, sedentary behavior, and light-intensity physical activity, but systematically overestimated step count, sedentary behavior, and vigorous-intensity physical activity, with poor agreement across all indicators.
• Wear compliance was higher for the wrist-worn activity trackers than for the waist-worn accelerometer under free-living conditions in Japanese school-aged children.

**What are the implication of the main findings?**

• Wrist-worn activity trackers may be useful for describing general patterns of lower-intensity physical activity and sedentary behavior in children at the population level.
• Caution is required when using wrist-worn trackers for accurate individual-level physical activity assessment, particularly for higher-intensity activities.

**Abstract:**

**Background**: Wrist-worn consumer activity trackers are widely used to promote physical activity (PA) and reduce sedentary behavior (SB). However, evidence regarding their validity for measuring PA and SB in free-living school-aged children remains limited. This study evaluated the concurrent validity and wear compliance of a wrist-worn consumer activity tracker in school-aged children under free-living conditions with protocol-defined wear requirements. **Methods**: A total of 102 children (mean age: 10.2 years; 44.1% girls) wore a wrist-worn device (Fitbit Ace) and a waist-worn accelerometer (Omron Active Style Pro HJA-750c, ASP-750c). Of the 1122 person-days collected over 11 days, 135 person-days meeting inclusion criteria for both devices were included (≥10 h/day wear time and an inter-device wear time difference of ≤60 min). Step count and time in SB, light (LPA), moderate (MPA), vigorous (VPA), and moderate-to-vigorous PA (MVPA) were assessed. Correlations, mean absolute percentage error (MAPE), agreement, and wear compliance between the two devices were examined. **Results**: Correlations were strong for step count (r = 0.86), SB (r = 0.72), and LPA (r = 0.71); however, agreement was poor, with systematic overestimation of step count, SB, VPA, and MVPA and underestimation of LPA and MPA by the Fitbit Ace, and MAPE exceeding 20% for all PA variables. Wear compliance (≥10 h/day on ≥4 days) was higher for the Fitbit Ace (97.0%) than for the ASP-750c (62.2%). **Conclusions**: Although the Fitbit Ace may be useful for characterizing general patterns of LPA and SB in school-aged children, caution is warranted for accurate individual-level PA assessment.

## 1. Introduction

Physical activity (PA) provides numerous health-related benefits [1], including enhancing cognitive skills and prosocial behavior among children and adolescents [2], with many of these benefits extending into adulthood [1,3]. Additionally, excessive sedentary behavior (SB) has been shown to increase the risk of multiple adverse health outcomes, especially among physically inactive populations [4]. Despite this evidence, 81% of adolescents aged 11–17 years are insufficiently physically active, with excessive sedentary time being widespread among children and adolescents globally [5]. Thus, urgent strengthening of policies and programs to effectively increase population PA levels among children and adolescents is needed [1].

To identify effective policies and programs for promoting PA and reducing SB, accurate measurement of activity is essential [6]. Doubly labeled water is widely regarded as the gold standard method for estimating free-living energy expenditure [7]; however, the high cost limits its practicality for large-scale surveillance and epidemiological studies [8]. Consequently, research-grade accelerometers are commonly used as a practical objective approach to quantify habitual PA in free-living conditions [9]. However, their use entails relatively high costs, low wearing compliance, and extensive training for data processing, and does not lead to the maintenance of long-term monitoring after studies are completed [10].

Consumer wearable devices—often referred to as “wearable activity trackers (e.g., smartwatches, wristbands, and accelerometers)”—are widely used in healthcare and wellness settings, offering continuous monitoring of various biometric parameters, such as PA, sleep patterns, and more [11]. Moreover, these devices not only monitor daily activity but also serve as motivational tools to encourage PA and reduce SB among children [12,13,14,15,16]. In addition, these devices are simple, inexpensive, and easy to use, making them particularly suitable for children and adolescents [15]. Thus, wearable activity tracker monitoring could present a promising option for increasing PA levels in this population. Particularly, wrist-worn activity trackers offer better compliance when compared with waist-worn devices because they are easy to wear, comfortable, and waterproof, reducing participant burden [15,17]. Hence, users may prefer to wear them continuously day and night [18].

Fitbit is a popular brand of commercial wearable activity trackers, and many studies have shown that the use of Fitbit devices in interventions may promote PA [19]. Systematic reviews have reported that Fitbit devices demonstrate consistently good accuracy for step counts, whereas their ability to provide accurate measurements for other physical activity metrics, including energy expenditure, is limited [20,21,22]. Moreover, the Fitbit Ace, which was developed to assess daily PA and SB in children aged 8 years and older, has not yet been adequately evaluated.

To our knowledge, the Fitbit Ace has only been evaluated in studies that have assessed step counts under experimental conditions (one used the Fitbit Ace 2, the successor to the Fitbit Ace) [23,24] and one study in which the researchers evaluated the validity of Fitbit Ace 2–derived step counts and moderate-to-vigorous PA (MVPA) under free-living conditions [25]. None of these studies adequately demonstrated the validity of estimating MVPA and step count. Moreover, no studies have investigated SB and light PA (LPA).

Furthermore, wrist-worn Fitbit devices have been shown to achieve higher wear compliance than waist-worn devices [26] and to facilitate extended monitoring under free-living conditions [27]. However, no study has examined the level of wear compliance among Japanese children. The widespread adoption of consumer-wearable activity devices with demonstrated validity and high wear compliance could facilitate the development of effective interventions to promote PA and reduce SB among schoolchildren [12,13,14,15,16].

Therefore, we aimed to evaluate the concurrent validity, including SB and LPA, and wear compliance of the Fitbit Ace for PA assessment under free-living conditions in school-aged children.

## 2. Materials and Methods

### 2.1. Participants

Participants were recruited from two of the nine schools that participated in the Koshu GRoup Activity, Active Play and Exercise study—a school-based cluster randomized controlled trial that was conducted from December 2018 to March 2020 [28]. We included 107 children in the fourth and fifth grades (aged 9–11 years) from the 2018 school year. Children who did not have any medical conditions that prohibited their participation in active play programs were eligible. Potential participants with anticipated long-term absence (≥30 days per year), for whom completion of the planned 11-day continuous measurement was considered difficult, were excluded.

The study protocol was approved by the Ethics Committee of the University of Yamanashi, the institution responsible for conducting the study (approval no. 1929), and by the Institutional Review Board of the School of Allied Health Sciences at Kitasato University, the authors’ affiliated institution at the time of the study (approval no. 2018-031). The study was conducted with the cooperation of the Health Promotion Division and the Board of Education of the Koshu City Administration Office. Ethical approval was obtained from the principals of the participating schools. All parents or legal guardians provided written informed consent for their children’s participation in the study, and informed assent was obtained from all participating children.

### 2.2. Sample Size

A sample size of 20 participants was determined a priori based on a power of 0.80, an alpha level of 0.05, and an expected strong correlation coefficient of r = 0.60 [29]. The sample size was calculated using Stata 18 SE (StataCorp LLC, College Station, TX, USA). Furthermore, we assumed that at least 64 children were needed, based on our previous studies that targeted children in Koshu City [28], considering the accept rate (90%), data missing due to invalid accelerometers (30% invalid), and sex-stratified analysis for wear compliance assessment. Because no single school had more than 64 children in the fourth and fifth grades, two large schools from different regions of Koshu City were selected. The combined number of children from these schools was 107, which exceeded the required sample size.

### 2.3. Instruments

#### 2.3.1. Fitbit Ace

The Fitbit Ace (Fitbit Inc., San Francisco, CA, USA) is a wrist-worn activity tracker (35 × 16 × 10 mm; 26 g including the band, size M) designed to monitor PA and sleep in children aged ≥ 8 years. It features a triaxial accelerometer and continually acquires data, with an onboard storage capacity for ~5 days without syncing.

#### 2.3.2. Omron Active Style Pro HJA-750c

The Omron Active Style Pro HJA-750c (ASP-750c; Omron Healthcare, Kyoto, Japan)—a waist-worn tri-axial accelerometer (52 × 40 × 12 mm; 23 g including battery)—was used as the reference device for measuring PA and SB. The Active Style Pro 350 IT (Omron Healthcare, Kyoto, Japan), using the same acceleration sensor and algorithm as the 750c [30], has been validated, and when compared with double labeled water measurements obtained under free-living conditions, this device showed a smaller difference (69 kcal/day) than the ActiGraph GT3X (Pensacola, FL, USA) (524.9 kcal/day), which was the smallest difference among the devices examined [31]. The ASP-750c estimated substantially more daily time spent in moderate-to-vigorous PA but showed similar daily step counts compared with ActiGraph in free-living Japanese adults [32] and children [33].

#### 2.3.3. Procedure

The participants were instructed to wear the ASP-750c and Fitbit Ace simultaneously for 11 days (4 weekend days [2 weekends] and 7 weekdays [5 days between 2 weekends, and 1 day before and after 2 weekends]). The ASP-750c was worn on the right side of the waist from wake-up to bedtime, except during water-based activities (e.g., bathing or swimming) and activities associated with a high risk of injury. The Fitbit Ace was worn on the nondominant wrist continuously, including during sleep (unless it interfered with activities or posed a safety risk), to reduce the misclassification of sleep as sedentary time and thereby improve the accuracy of sedentary time estimation.

The participants’ ages, heights, and weights were collected via physical measurements taken during medical checkups conducted at elementary schools in January 2019. Body mass index (kg/m^2^) was calculated using height and weight, and obesity was categorized using the study software of the Joint Committee on Standard Values of the Japan Pediatric Endocrine Society and Japan Society for Growth Research [34].

#### 2.3.4. Data Processing

The ASP-750c CSV data files were downloaded using the Omron health management software BI-LINK (Professional Edition version 1.0). Because the ASP-750c prediction equations were developed for adults, metabolic equivalent (MET) values may be overestimated when applied to elementary school children [35]. Therefore, in accordance with previous studies [35], we converted the MET values using a child-specific conversion equation recommended for this age group, specifically a quadratic polynomial equation that accounts for all activities. A macro program (ver. 1.1; Japan Physical Activity Research Platform) [36] was used for CSV data processing. Data were collected in 1 min epochs. Activity behaviors were categorized by intensity based on the METs: SB (≤1.5 METs), LPA (>1.5 and <3.0 METs), moderate PA (MPA, >3.0 and <6.0 METs), and vigorous PA (VPA, ≥6.0 METs).

The researcher created a Fitbit account for each participant, and pre-configured devices were distributed to the children. Data from the Fitbit Ace are automatically stored on the Fitbit server when synchronized via Bluetooth. However, because the children could not use the Fitbit app at school and the built-in device can only store 5 days of measurement data, the researchers visited the school once every 3 days during the 11-day measurement period and used their own smartphones/tablets with the Fitbit app (Fitbit Inc., San Francisco, CA, USA) to synchronize and obtain the data. Data were transferred via Bluetooth to the Fitbit application programming interface (API) using the Fitbit mobile app. TechDoctor Inc. (Tokyo, Japan) retrieved and downloaded the data using the Fitbit API. A Python (3.10.2) script was used to collect the data in 1 min intervals and aggregate it. Although the algorithms used to determine PA intensity are proprietary, “Sedentary,” “Lightly active,” “Moderately/Fairly active,” and “Very active” activity intensity levels from Fitbit were considered sedentary (≤1.5 METs), light (>1.5 and <3.0 METs), moderate (>3.0 and <6.0 METs), and vigorous (≥6.0 METs) activity, respectively [37].

#### 2.3.5. Validity Assessment

For both devices, wear time used for evaluation was defined consistently as the period from wake-up to bedtime. Participants were instructed to wear the Fitbit Ace during sleep; therefore, when sleep duration was recorded, wear time was defined as the interval from wake-up to bedtime. When sleep duration was not recorded, sleep–wake times were assumed based on a previous study of schoolchildren in Koshu City [38]: wake-up times were set at 6:30 on weekdays and 7:00 on weekends, and bedtimes were set at 21:30 on weekdays and 22:00 on the night preceding weekends. We confirmed that the wake-up and bedtimes observed in the raw data from the ASP-750c were generally consistent with these assumed time windows. For both devices, non-wear time was defined as periods of at least 60 consecutive minutes with no accelerometer signal. Daily wear time was calculated by subtracting non-wear time and sleep duration from the 24 h period.

We defined valid person-days based on whether both devices had valid days (≥10 h of wear). Because previous studies have reported that excluding data with a wear time difference of ≥60 min between devices improves the accuracy of classifying achievement versus non-achievement of step-count thresholds [39], we restricted the analytic sample to person-days in which the difference in wear time between the two devices was less than 60 min [40], in order to ensure more accurate device comparisons. We evaluated the step count, time spent in each PA intensity (LPA, MPA, VPA, and MVPA), and SB on valid person-days.

#### 2.3.6. Wear Compliance Assessment

The primary wear-time outcome was the proportion of children who achieved a wear time of ≥10 h per day for at least 4 days during the 11-day monitoring period [41]. In addition, we evaluated alternative wear-time criteria by calculating the proportion of person-days meeting thresholds of ≥8, ≥12, and ≥14 h per day. We also examined the number of weekdays and weekend days with ≥10 h of valid wear time and calculated the proportion of children who met the following criteria: ≥2 valid days regardless of weekday or weekend; ≥3 valid days (≥2 weekdays and ≥1 weekend day); ≥5 valid days (≥4 weekdays and ≥1 weekend day, or ≥3 weekdays and ≥2 weekend days); ≥6 valid days (≥5 weekdays and ≥1 weekend day, or ≥4 weekdays and ≥2 weekend days); and ≥7 valid days (≥6 weekdays and ≥1 weekend day, or ≥5 weekdays and ≥2 weekend days). As a supplementary analysis, these wear-time outcomes were examined stratified by sex.

### 2.4. Statistical Analysis

#### 2.4.1. Validity Analyses

Concurrent validity was determined using Pearson’s correlations between step counts, PA, and SB estimates from the ASP-750c and Fitbit Ace. Because MPA, VPA, and MVPA had skewed distributions with a long right tail, Spearman’s rank correlation was adopted. The following cut-off values were used to interpret the Pearson correlations: *r* < 0.20 = very weak, 0.20–0.39 = weak, 0.40–0.59 = moderate, 0.60–0.79 = strong, and 0.80–1.0 = very strong [42].

Agreement was assessed using the intraclass correlation coefficient (ICC; 2-way random, absolute agreement). The cut-off values used to interpret the ICC were <0.20 = poor, 0.21–0.40 = fair, 0.41–0.60 = moderate, 0.61–0.80 = substantial, and 0.81–1.00 = almost perfect [43]. Bland–Altman plots were used to visualize the agreement between the ASP-750c and Fitbit Ace. Proportional bias was assessed by examining the linear association between the measurements of the two devices. Additionally, a paired Student’s t-test was used to compare the differences in measurements between the two devices within the same participants. The mean absolute percentage error (MAPE) was calculated as follows to evaluate the similarity of the estimates: the absolute difference between the ASP-750c and Fitbit Ace values was divided by the ASP-750c value (result expressed in percentage), which was averaged across all data points. The MAPE was categorized based on commonly used accuracy cutoffs for measuring step count with wearable devices—with an acceptable error of ±10% in free-living settings [44]—as poor (>20%), moderate (10.1–20%), good (3.1–10%), and excellent (0–3%).

#### 2.4.2. Wear-Compliance Analyses

A Student’s t-test was used to compare the mean wearing time throughout the measurement period between the ASP-750c and the Fitbit Ace. The percentages that met the wear time per person-day (8 h, 10 h, 12 h, and 14 h) and those that met the reasonable number of days required to estimate habitual PA (2–7 days) were compared using chi-square tests.

All statistical analyses were performed using STATA 18 SE (StataCorp LLC, TX, USA). Statistical significance was defined as *p* < 0.05 for all analyses.

## 3. Results

### 3.1. Participants and Valid Measurement Person-Days

Overall, 102 children agreed to participate (consent rate: 95.3%). Their characteristics are presented in Table 1; the average age was 10.2 years (SD = 0.7), and 44.1% were girls.

**Figure 1:**
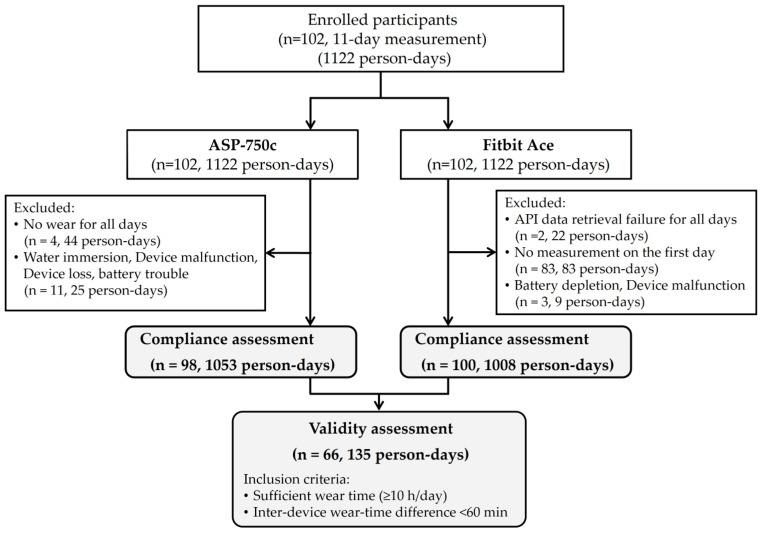
Flowchart of participant inclusion and device-specific data processing.

A total of 1122 person-days of measurements were obtained from the 102 participants over the 11-day measurement period (Figure 1). For the Fitbit Ace, data from 83 children were not recorded on the first day (83 person-days). In addition, data from three children (9 person-days) were excluded because of battery depletion or device malfunction, and data from two children (22 person-days) were excluded because data could not be retrieved via the API for unknown reasons. As a result, data from 100 children comprising 1008 person-days (89.8% of all measured person-days) were included in the wear-compliance assessment. For the ASP-750c, data from four children who did not wear the device on any of the 11 days (44 person-days) were excluded. In addition, data from 11 children (25 person-days) were excluded due to device-related problems (e.g., water immersion, malfunction, device loss, and battery trouble). Consequently, data from 98 children comprising 1053 person-days (93.9%) were included in the wear-compliance assessment.

For the validity analysis, a subset of 135 person-days (12.0% of all measured person-days) was selected from the total dataset based on the predefined criteria (≥10 h/day wear time for both devices and an inter-device wear-time difference of <60 min). Among these, sleep duration measured by the Fitbit Ace was available for 114 person-days (84.4%), and estimated sleep duration was applied for 21 person-days.

### 3.2. Concurrent Validity

For the 135 person-days included in the analysis, the mean wear time was 837.4 min (standard deviation [SD]: 84.9) for the ASP-750c and 856.6 min (SD: 83.1) for the Fitbit Ace.

A very strong positive correlation between the two devices was found for step count (r = 0.86, *p* < 0.001). Strong positive correlations were also found for SB and LPA. The correlations were moderate for MPA, VPA, and MVPA (Table 2).

Table 3 shows the differences between the values obtained using the two devices; significant differences were found for all PA variables. The Fitbit Ace recorded higher values for step count, SB, VPA, and MVPA, and lower values for LPA and MPA. A significant difference was observed in the recording of VPA between the two devices. The MAPE exceeded 20% for all the variables: the smallest value was found for step count (approximately 21%), and the highest for MVPA (120%). For VPA, the MAPE could not be calculated given the inclusion of zeros.

**Figure 2:**
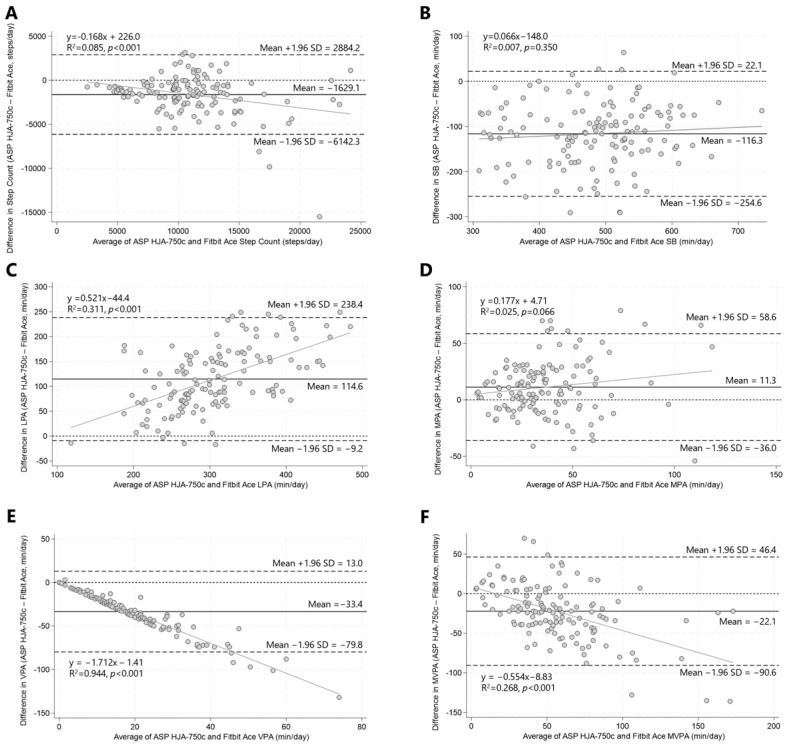
Bland–Altman plots illustrating agreement between ASP-750c and Fitbit Ace–derived (**A**) step counts, (**B**) SB, (**C**) LPA, (**D**) MPA, (**E**) VPA, and (**F**) MVPA. The solid line in the center shows the mean difference (bias) between the devices, and the dotted lines at the top and bottom indicate the upper (+1.96 SD) and lower (−1.96 SD) limits of agreement. ASP: Active Style Pro; SB: sedentary behavior; LPA: light physical activity; MPA: moderate physical activity; VPA: vigorous physical activity; MVPA: moderate-to-vigorous physical activity.

Figure 2 shows the agreement between the two devices using a Bland–Altman plot. The Fitbit Ace overestimated the step count, SB, VPA, and MVPA compared to the ASP-750c, whereas it underestimated LPA and MPA. Substantial discrepancies were observed in SB, LPA, and VPA measurements. The majority of plots for SB and VPA fell below zero, which indicates that the Fitbit Ace systematically overestimated these variables compared to the ASP-750c. Conversely, most LPA plots were positioned above zero, which shows that the Fitbit Ace consistently underestimated LPA relative to the ASP-750c.

The regression analysis revealed that proportional errors were observed in the step count, LPA, VPA, and MVPA, where the larger the value, the greater the discrepancy (in the negative direction for LPA; in the positive direction for VPA and MVPA). In particular, although a high correlation was observed for VPA between the two devices, the agreement was low, and the limits of agreement were wide. Overall, across the PA variables examined, wide limits of agreement were observed even when correlations between the two devices were present.

### 3.3. Wear Compliance

The average daily wearing time was 9.4 h/person-day for the ASP-750c and 11.9 h/person-day for the Fitbit Ace. The Fitbit Ace was worn significantly longer overall and by both sexes (Table 4). Using 8 h, 10 h, 12 h, and 14 h of wear time as the criteria for person-days, the Fitbit Ace compliance was higher than the ASP-750c compliance for all wear-time criteria. This association remained the same when the data for boys and girls were examined separately (Supplementary Table S1).

Significantly more children met the criteria for ≥10 h of wear time per day and ≥4 valid days for the Fitbit Ace (97%) than for the ASP-750c (62.2%) (Table 5). Although no difference was observed between devices for ≥2 valid wear days, the Fitbit Ace consistently exhibited higher wear compliance than the ASP-750c for the remaining criteria. In sex-stratified analyses, the Fitbit Ace demonstrated higher wear compliance than the ASP-750c across all criteria in boys, and across all criteria except ≥2 valid wear days in girls (Supplementary Table S2).

## 4. Discussion

This study demonstrated that Fitbit Ace measurements were strongly correlated with step count, SB, and LPA, and moderately correlated with MPA, VPA, and MVPA compared with the ASP-750c in school-aged children in free-living conditions. However, the agreement between the two devices was low, and the MAPE compared to the ASP-750c exceeded 20% for all indicators, especially for MVPA (120%). Additionally, the Fitbit Ace overestimated step count, SB, VPA, and MVPA, and underestimated LPA and MPA compared to the ASP-750c. Proportional errors were identified for step count, LPA, VPA, and MVPA, with larger errors for larger values. In contrast, wear compliance was consistently higher for the Fitbit Ace than for the waist-worn ASP-750c across all measured activity indicators.

### 4.1. Validity Findings

The step count recorded by the Fitbit Ace was strongly correlated with and overestimated by the waist-worn accelerometer. This finding is consistent with those of a previous study in adults, which reported that consumer model trackers such as Fitbit are the most accurate in measuring step count but have insufficient validity for measuring MVPA [20,21,22,45]. However, our results contradict those of a study conducted in children in which the same Fitbit Ace was used and the step count was underestimated [23]. This discrepancy is likely attributable to differences in the reference standards used. In our study, step counts were compared against a waist-worn device, whereas previous studies used direct observation as the gold standard. Wrist-worn devices have been shown to record higher step counts than waist-worn devices [46], while accelerometer-based step counts tend to be underestimated relative to direct observation [47]. In addition, the ASP-750c has been reported to underestimate step counts in Japanese children [48]. Together, these factors may have amplified the relative overestimation of step counts observed for the Fitbit Ace in this study.

The results for MPA, VPA, and MVPA revealed moderate correlations but low agreement. These findings are consistent with those of previous studies on wrist-worn wearable devices [45] and the successor of the Fitbit Ace, the Fitbit Ace 2 [24]. In particular, MVPA showed the largest relative error, as reflected by the highest MAPE value, indicating substantial discrepancies between devices, which aligns with the findings of a previous study comparing data from the Fitbit Flex 2 (an activity tracker with functionality similar to the Fitbit Ace) with measurements obtained from the waist-worn ActiGraph GT9X accelerometer [49]. Wrist-worn devices are more likely to classify non-ambulatory activities involving arm or upper-body movements, such as hand swinging, as MVPA or VPA, even when the actual energy expenditure is relatively low [50], which may have contributed to the observed overestimation.

Conversely, the results for LPA differed from those of our study, showing low correlation and agreement [49]. The different validity criteria used in these studies (e.g., wearing time > 8 h) may have contributed to these discrepancies.

For SB, Byun et al. [51] reported a strong correlation (*r* = 0.87, Pate’s criterion; *r* = 0.85, Everson’s criterion), similar to our findings. By contrast, Schmidt et al. [49] reported moderate correlations. These discrepancies may also be attributable to differences in the criteria for wearing time. In our study, the wear-time difference between the two devices had to be ≤60 min. However, Byun et al. aligned their data in 1 min increments, ensuring no wear-time difference [51]. Comparatively, Schmidt et al. included data when both devices were worn for ≥8 h per day [49], which may have contributed to the weaker correlation. Although Fitbit activity trackers (e.g., Fitbit Flex, Fitbit Charge HR) may tend to overestimate SB, prior studies have demonstrated high classification accuracy, supporting their validity for SB assessment in children and adolescents [52].

The Fitbit Ace tended to show relatively smaller discrepancies for step counts and lower-intensity activities, such as LPA and SB, compared with higher-intensity PA; however, MAPE values exceeded acceptable thresholds across all intensity variables except VPA (not computable due to zeros), indicating limited overall accuracy.

### 4.2. Wear Compliance Findings

Herein, the widely used validity criterion of ≥10 h wear time per day for 4 days (including 1 weekend day) was met in 97% of cases for the Fitbit Ace compared to 62% for the ASP-750c.

This finding is consistent with previous studies using the adult wrist-worn Fitbit Charge HR [53] and other validated wrist-worn accelerometers (ActiGraph [54], GENEActive [55]), and was also observed in the present study of Japanese children using a consumer-grade wrist-worn device. Furthermore, approximately 80% of the children met the requirement of wearing the Fitbit Ace for at least 7 days (5 weekdays and 2 weekend days, representing typical PA for a week). This suggests that the Fitbit Ace is a suitable device for long-term measurement of daily PA among school-aged children.

However, the percentage of valid data based on daily wear time decreased to 73% and nearly 50% when the validity criterion was set at ≥12 h and ≥14 h, respectively. Wing et al. [53] also reported that the proportion of valid data decreased as the criteria for daily wear-time increased (73% at 10 h, 58% at 12.5 h, and 21% at 15 h). Given that high compliance allows for the measurement of validated daily activity over a shorter period and enhances confidence in the representativeness of daily PA [56], our findings suggest that the Fitbit Ace may be useful for long-term activity measurement in school-aged children. On the other hand, although these issues were unlikely to have a substantial impact on the validity assessment, several practical challenges associated with the Fitbit Ace were identified in comparison with the Omron device, including the need for regular synchronization with the smartphone application, more frequent battery charging over short intervals, and difficulties in retrieving data via the API.

### 4.3. Strengths and Limitations

This is the first study to investigate the validity of all PA intensities, including SB and LPA, and the wear compliance of the Fitbit Ace under free-living conditions over an 11-day measurement period, including 2 weekends simultaneously, with a sufficient sample of children. This study provides additional evidence that helps address existing gaps in the literature and suggests that wrist-worn wearable devices, such as the Fitbit Ace, may have potential utility for assessing lower-intensity PA and SB for longer periods in epidemiological studies of children.

However, this study has some limitations. First, because one of the objectives was to examine compliance with wrist-worn and waist-worn accelerometers, the waist-worn accelerometer was used as the gold standard device. Future studies should evaluate validity by comparing wrist-worn devices with accelerometers that have higher established validity, such as ActiGraph, as well as with gold-standard methods, including doubly labeled water, ideally in combination with activity logs to characterize activity types when assessing measurement accuracy [57]. Second, as this study assessed the validity of a wrist-worn Fitbit Ace using a waist-worn ASP-750c as the reference, the results should be interpreted as reflecting both device-specific characteristics and wear-location effects. Differences in sensor sensitivity and motion detection across wear locations may contribute to these findings. In particular, wrist-worn devices are more sensitive to upper-extremity movements and have been shown to misclassify light-intensity activities involving substantial arm movements as MVPA, thereby tending to overestimate MVPA compared with waist-worn devices [50]. While wrist-to-waist conversion equations have been proposed in previous studies [58], their application is constrained by the lack of access to raw acceleration data from Fitbit devices. Accordingly, further validation studies using research-grade accelerometers (e.g., ActiGraph) worn at a common site (the wrist) are warranted. Third, participants were instructed to wear the ASP-750c only during waking hours, while the Fitbit Ace was worn continuously, including during sleep. Because this difference in wear protocols may have influenced wear compliance, future studies should compare devices under equivalent conditions. Fourth, in this validity study, analyses were restricted to person-days in which the difference in wear time between the two devices was less than 60 min. Consequently, the resulting dataset may be subject to selection bias, as it is likely to overrepresent participants who were more compliant with device wear. However, from the perspective of device validation, comparable wear time between devices is a fundamental prerequisite for fair assessment of measurement agreement [59]. Nevertheless, because comparable wear time between devices is a fundamental prerequisite for device validation, this criterion ensures that a fair comparison between the devices is maintained. Fifth, although a 1 min epoch length was used in this study, shorter (1–15 s) lengths are recommended for children because the time spent on MVPA progressively decreases as the epoch length increases [60]. Therefore, our results should be confirmed using shorter epoch lengths. Sixth, although our analysis was limited to measurement days with wear-time differences of ≤60 min between the two devices, wearing time may considerably influence PA and SB estimates. Future studies should use wearable devices with heart rate monitors, as more detailed second-by-second wearing time can be confirmed by incorporating captured heart rate data [40]. Finally, the Fitbit Ace used in this study has been discontinued, and successor models (Fitbit Ace 2 and Ace 3) have already been released. Because these successor models may differ in algorithm and wearability, the extent to which the present findings can be generalized to newer models or other wearable devices remains uncertain. Although further research is required to establish the accuracy and precision of PA estimates from the Fitbit devices, the findings of this study provide useful evidence for interpreting PA and SB data collected using the original Fitbit Ace devices that remain in use.

## 5. Conclusions

Although the Fitbit Ace demonstrated higher wear compliance and stronger correlations for step count, SB, and LPA, agreement with a waist-worn accelerometer was low, particularly across all PA variables. Thus, although the Fitbit Ace may be useful for characterizing general patterns of LPA and SB in children, caution is warranted when using the device for more accurate individual-level PA assessment.

## Figures and Tables

**Table 1:** Participant characteristics by sex among Japanese school-aged children.

	Total		Boys		Girls	
	n = 102		n = 57		n = 45	
Age, years (SD)	10.2	(0.7)	10.1	(0.7)	10.2	(0.6)
Height, cm (SD)	140.1	(7.2)	139.2	(7.2)	141.3	(7.3)
Weight, kg (SD)	35.5	(7.7)	35.6	(8.7)	35.3	(6.4)
BMI, kg/m^2^ (SD)	17.9	(2.6)	18.2	(3.0)	17.6	(2.1)
Weight status, n (%)						
Underweight	2	(2.0)	1	(1.8)	1	(2.2)
Healthy weight	84	(82.3)	44	(77.2)	40	(88.9)
Overweight	12	(11.8)	8	(14.0)	4	(8.9)
Obese	4	(3.9)	4	(7.0)	0	(0.0)

**Table 2:** Concurrent validity based on Pearson’s correlation coefficients between the ASP-750c and the Fitbit Ace.

	Correlation		
	(Person-Days = 135)		
	*r*	95% CI	*p*-Value
Steps	0.86	0.80–0.90	<0.001
SB	0.72	0.63–0.79	<0.001
LPA	0.71	0.62–0.79	<0.001
MPA	0.48	0.38–0.60	<0.001 *
VPA	0.44	0.30–0.57	<0.001 *
MVPA	0.52	0.39–0.66	<0.001 *

* Spearman’s rank correlation. SB: sedentary behavior; LPA: light physical activity; MPA: moderate physical activity; VPA: vigorous physical activity; MVPA: moderate to vigorous physical activity.

**Table 3:** Mean absolute percentage errors and intraclass correlation coefficient of steps, SB, and each PA intensity level between the ASP-750c and the Fitbit Ace.

	Person–Days = 135										
	ASP-750c		Fitbit Ace		Mean Difference		95% CI	*p*-Value	MAPE, % (SD)		ICC (95% CI)
Steps, step (SD)	10,202.3	(3812.8)	11,831.3	(4459.6)	−1629.1	(2302.7)	(−2021.0–−1237.1)	<0.001	21.7	(18.6)	0.79 (0.49–0.89)
SB, min (SD)	425.5	(96.6)	541.8	(91.3)	−116.3	(70.6)	(−128.3–−104.3)	<0.001	31.2	(22.3)	0.41 (−0.10–0.72)
LPA, min (SD)	362.6	(89.2)	248.0	(56.5)	114.6	(63.2)	(103.8–−125.4)	<0.001	30.3	(13.0)	0.30 (−0.09–0.62)
MPA, min (SD)	42.8	(26.3)	31.5	(23.0)	11.3	(24.1)	(7.2–15.4)	<0.001	61.9	(88.1)	0.48 (0.28–0.62)
VPA, min (SD)	2.0	(3.4)	35.4	(25.1)	−33.4	(23.7)	(−37.4–−29.4)	<0.001	—	—	0.05 (−0.05–0.16)
MVPA, min (SD)	44.8	(27.9)	66.9	(44.3)	−22.1	(35.0)	(−28.1–−16.2)	<0.001	120.7	(220.5)	0.47 (0.19–0.65)

ASP: Active Style Pro; MAPE: mean absolute percentage errors; ICC: intraclass correlation coefficient. SB: sedentary behavior; LPA: light physical activity; MPA: moderate physical activity; VPA: vigorous physical activity; MVPA: moderate to vigorous physical activity.

**Table 4:** Comparison of valid wear hours between the ASP-750c and the Fitbit Ace.

	ASP-750c		Fitbit Ace		*p*-Value
	(Person-Days = 1053)		(Person-Days = 1008)		
Total wear time, hours/person-days (SD)	9.4	(5.9)	11.9	(4.7)	<0.001
Valid wear hours, person-days (%)					
≥8 h	693	(65.8)	902	(89.5)	<0.001
≥10 h	609	(57.8)	839	(83.2)	<0.001
≥12 h	463	(44.0)	738	(73.2)	<0.001
≥14 h	232	(22.3)	515	(51.1)	<0.001

ASP: Active Style Pro.

**Table 5:** Comparison of valid wear days (defined as ≥10 h of wear time per day) between the ASP-750c and the Fitbit Ace.

	ASP-750c		Fitbit Ace		*p*-Value
	(n = 98)		(n = 100)		
Valid wear days, n (%)					
≥2 days	90	(91.8)	99	(99.0)	0.016
≥3 days (2 WD and 1 WE)	64	(65.3)	98	(98.0)	<0.001
≥4 days (3 WD and 1 WE)	61	(62.2)	97	(97.0)	<0.001
≥5 days (4 WD and 1 WE)	58	(59.2)	94	(94.0)	<0.001
≥5 days (3 WD and 2 WE)	45	(45.9)	89	(89.0)	<0.001
≥6 days (5 WD and 1 WE)	48	(49.0)	85	(85.0)	<0.001
≥6 days (4 WD and 2 WE)	43	(43.9)	87	(87.0)	<0.001
≥7 days (6 WD and 1 WE)	35	(35.7)	72	(72.0)	<0.001
≥7 days (5 WD and 2 WE)	36	(36.7)	79	(79.0)	<0.001

ASP: Active Style Pro; WD: weekdays; WE: weekend days. A valid wear day was defined as ≥10 h of wear time per day.

## Data Availability

The data presented in this study are unavailable due to privacy or ethical restrictions. The data are not publicly available due to ethical standards.

## Supplementary Materials

The following supporting information can be downloaded at: https://www.mdpi.com/article/10.3390/children13020184/s1. Table S1: Comparison of valid wear hours between the ASP-750c and Fitbit Ace by sex. Table S2: Comparison of valid wear days between the ASP-750c and Fitbit Ace by sex.

## Abbreviations

The following abbreviations are used in this manuscript:

PA	Physical Activity
SB	Sedentary Behavior
MVPA	Moderate-to-Vigorous Physical Activity
LPA	light Physical Activity
ASP-750c	The Omron Active Style Pro HJA-750c
MET	Metabolic Equivalent
MPA	Moderate Physical Activity
API	Application Programming Interface
ICC	Intraclass Correlation Coefficient
MAPE	The Mean Absolute Percentage Error
