# Supplementary material for: Validity and Wear Compliance of Wrist-Worn Consumer Activity Trackers Among Japanese School-Aged Children Under Free-Living Conditions
Source: Children (Basel). 2026 Jan 28;13(2):184. doi: 10.3390/children13020184 (PMC12939141; doi:10.3390/children13020184)
Supplement: Supplementary file 1 [file children-13-00184-s001.zip › children-4095067-supplementary.pdf]

Table S1. Comparison of valid wear hours between the ASP-750c and Fitbit Ace by sex

	Boys			Girls		
	ASP-750c (Person-days = 581)	Fitbit Ace (Person-days = 561)	p-value	ASP-750c (Person-days = 472)	Fitbit Ace (Person-days = 447)	p-value
**Total wear time, min/person-days (SD)**	9.4 (6.0)	11.6 (4.9)	<0.001	9.3 (5.8)	12.3 (4.5)	<0.001
**Valid wear hours, person-days (%)**						
≥8 h	377 (64.9)	492 (87.7)	<0.001	316 (67.0)	410 (91.7)	<0.001
≥10 h	330 (56.8)	458 (81.6)	<0.001	279 (59.1)	381 (85.2)	<0.001
≥12 h	258 (44.4)	398 (70.9)	<0.001	205 (43.4)	340 (76.1)	<0.001
≥14 h	130 (22.4)	269 (48.0)	<0.001	102 (21.6)	246 (55.0)	<0.001

ASP: Active style Pro; WD: weekdays; WE: weekend day

Table S2. Comparison of valid wear days between the ASP-750c and Fitbit Ace by sex.

	Boys			Girls		
	ASP-750c (n = 54)	Fitbit Ace (n = 56)	p-value	ASP-750c (n = 44)	Fitbit Ace (n = 44)	p-value
**Valid wear days, n (%)**						
≥2 days	48 (88.9)	56 (100.0)	0.010	42 (95.5)	43 (97.7)	0.557
≥3 days (2 WD and 1 WE)	35 (64.8)	55 (98.2)	<0.001	29 (65.9)	43 (97.7)	<0.001
≥4 days (3 WD and 1 WE)	33 (61.1)	55 (98.2)	<0.001	28 (63.6)	42 (95.5)	<0.001
≥5 days (4 WD and 1 WE)	31 (54.4)	53 (93.0)	<0.001	27 (60.0)	41 (91.1)	0.001
≥5 days (3 WD and 2 WE)	23 (40.4)	49 (86.0)	<0.001	22 (48.9)	40 (88.9)	<0.001
≥6 days (5 WD and 1 WE)	26 (45.6)	47 (82.5)	<0.001	22 (48.9)	38 (84.4)	<0.001
≥6 days (4 WD and 2 WE)	22 (38.6)	47 (82.5)	<0.001	21 (46.7)	40 (88.9)	<0.001
≥7 days (6 WD and 1 WE)	18 (31.6)	36 (63.2)	0.001	17 (37.8)	36 (80.0)	<0.001
≥7 days (5 WD and 2 WE)	19 (33.3)	42 (73.7)	<0.001	17 (37.8)	37 (82.2)	<0.001

ASP: Active style Pro; WD: weekdays; WE: weekend day
